# Supplementary figures and images for: Local and landscape-level diversity effects on forest functioning
Source: PLoS One. 2020 May 14;15(5):e0233104. doi: 10.1371/journal.pone.0233104 (PMC7224498; doi:10.1371/journal.pone.0233104)

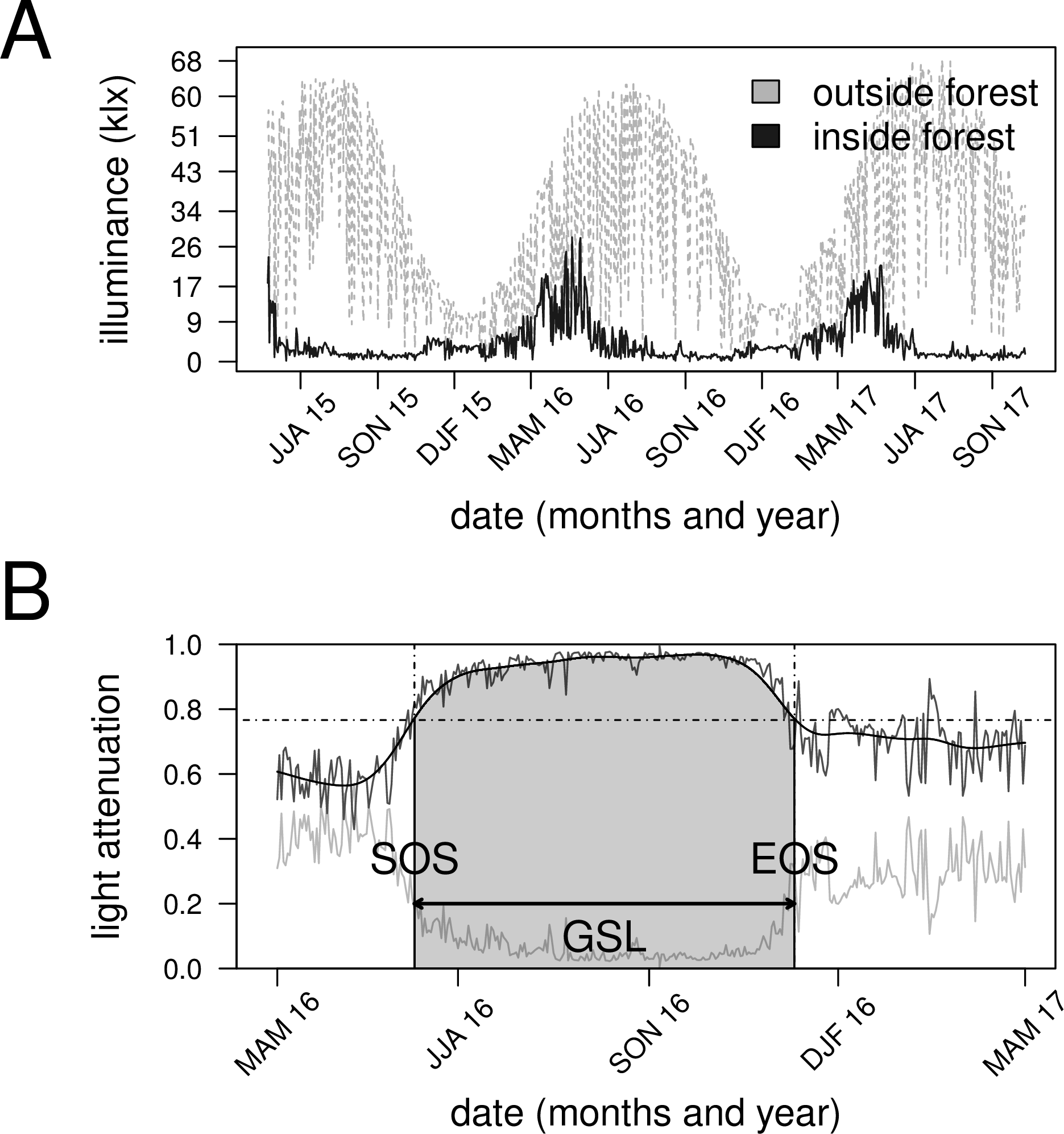

Supplement: S1 Fig — A: Temporal development of average daily illuminance (kilolux; i.e. klx) measured inside (dark grey) and outside (light grey) forest stands using corresponding sensors (HOBO UA-002-64; Onset Computer Corporation, Bourne, MA) from spring 2015 (March, April, May; MAM) until autumn 2017 (September, October, November; SON). B: Light attenuation by the forest canopy derived from the smoothed ratio of average daily (i.e. between 11.00 am and 4.00 pm) inside and outside forest illuminances (light grey) subtracted from 1 (dark grey). We defined yearly start of the season (SOS) as the day of the year when light attenuation first exceeded the mean of its annual minimum and maximum value (horizontal dashed line). Similarly, we derived the yearly end of the season (EOS) as the last day of the year before light attenuation fell below this threshold. We then calculated yearly growing-season length (GSL) as the number of days between SOS and EOS and finally averaged GSL across the years 2015 and 2016. We derived these metrics only for forests with a clear seasonal pattern in light attenuation, which we determined by applying the following restrictions: A minimum GSL of 60 days, a minimum growing season amplitude of 0.045 and a maximum number of 2 periods of consecutive days above the mean of annual minimum and maximum light attenuation, with a maximum number of 8 days in between these periods. In total, we obtained GSL values for a subset of 22 out of the 36 forest sites. (TIF) [file pone.0233104.s001.tif]

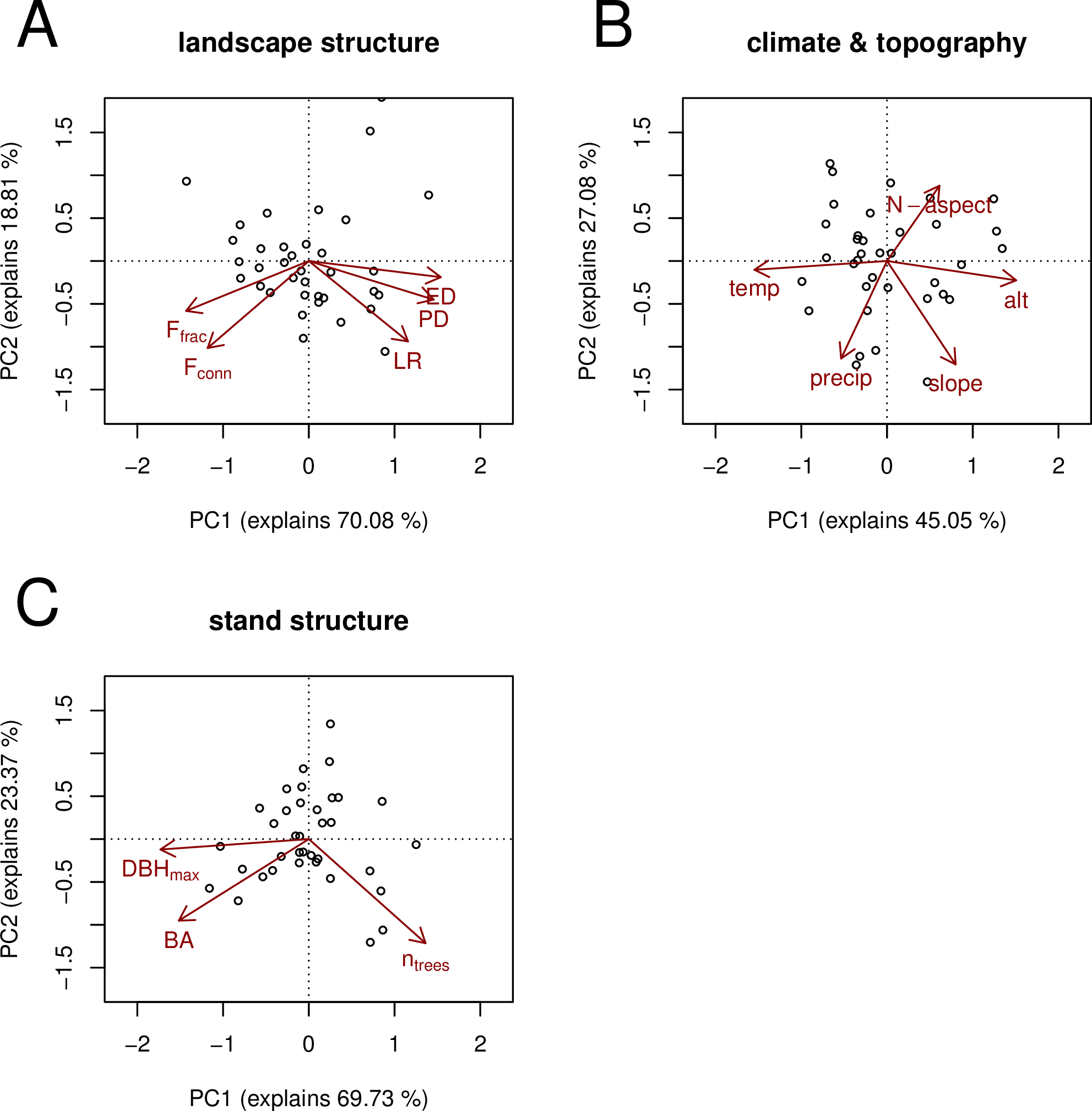

Supplement: S2 Fig — Principal component analyses (PCA) of variables related to landscape structure (A), climate and topography (B) and local stand structure (C). A: Edge density (ED; total length of borders between different land-cover types divided by the total landscape area), patch density (PD; the number of different land-cover patches divided by the total area of the landscape) and land cover richness (LR; the number of different land-cover types) closely clustered together and strongly differed in their loadings compared to the fraction of forest cover (Ffrac) and the connectivity of forest patches (Fconn) for the first principal component (PC1). B: Temperature (temp) and altitude (alt) showed the strongest negative covariance in loadings for PC1, indicating a strong temperature-altitude gradient in our dataset. We identified a second important gradient defined by precipitation (precip) that positively co-varied with slope and negatively co-varied with the northerly aspect (N-aspect) in their loadings for the second principal component (PC2). C: Our proxy for stand age and demographic structure DBHmax (average diameter at breast height of the three largest trees) clustered closely with cumulative basal area (BA) and both of these measures co-varied negatively with the number of trees (ntrees) in their loadings for PC1 (TIF) [file pone.0233104.s002.tif]
