# Supplementary material for: Local and landscape-level diversity effects on forest functioning
Source: PLoS One. 2020 May 14;15(5):e0233104. doi: 10.1371/journal.pone.0233104 (PMC7224498; doi:10.1371/journal.pone.0233104)
Supplement: S1 Table — We used information on the typical species composition of the 30 main Swiss forest types [34] to associate 1416 sites of the Swiss biodiversity monitoring program (BDM; biodiversitymonitoring.ch; Fig 1) with a likelihood of being a i) coniferous, ii) broadleaf or iii) mixed forest by using presence data of vascular plant species (Z9 Indicator of the BDM) and Jaccard’s index of similarity. (DOCX) [file pone.0233104.s003.docx]

|  |  |  |
| --- | --- | --- |
| **Forest type** | **Community type following [34]** | **Characteristic species** |
|  |  |  |
| coniferous | Molinio-Pinion | ***Calamagrostis varia, Molinia arundinacea, Pinus sylvestris,*** *Aster amellus, Cirsium tuberosum, Thlaspi montanum, Acer opalus, Anthericum ramosum, Aquilegia atrata, Aquilegia vulgaris, Aster bellidiastrum, Berberis vulgaris, Brachypodium pinnatum, Carex flacca, Carex montana, Carlina vulgaris, Convallaria majalis, Epipactis atrorubens, Festuca amethystine, Gymnadenia odoratissima, Helleborus foetidus, Laserpitium latifolium, Ligustrum vulgare, Lonicera alpigena, Melittis melissophyllum, Mercurialis perennis, Ophrys insectifera, Polygala amarelle, Rhamnus alpine, Taxus baccata, Viburnum lantana* |
|  | Erico-Pinion sylvestris | ***Carex alba****,* ***Erica carnea****,* ***Picea abies****,* ***Pinus sylvestris****, Cytisus nigricans, Daphne cneorum, Leontodon incanus, Rhamnus saxatilis, Thesium rostratum, Arctostaphylos uva-ursi, Brachypodium pinnatum, Carduus defloratus, Chamaecytisus supinus, Coronilla vaginalis, Daphne alpine, Dorycnium germanicum, Epipactis atrorubens, Goodyera repens, Hieracium pictum, Juniperus communis, Laserpitium gaudinii, Leucanthemum adustum, Monotropa hypopitys, Polygala chamaebuxus, Pyrola chlorantha, Pyrola rotundifolia, Teucrium chamaedrys, Vaccinium vitis-idaea, Viola collina, Viscum album* |
|  | Ononido-Pinion | ***Arctostaphylos uva-ursi****,* ***Carex humilis****,* ***Pinus sylvestris****, Astragalus exscapus, Astragalus monspessulanus, Coronilla minima, Odontites viscosus, Ononis rotundifolia, Antennaria dioica, Astragalus onobrychis, Avenella flexuosa, Carex halleriana, Cerastium arvense, Epipactis atrorubens, Erucastrum nasturtiifolium, Erysimum rhaeticum, Euphorbia seguieriana, Hippocrepis comosa, Minuartia laricifolia, Ononis pusilla, Peucedanum oreoselinum, Polygala chamaebuxus, Pyrola chlorantha, Saponaria ocymoides, Silene nutans, Teucrium chamaedrys, Thymus praecox, Vicia cracca, Viola rupestris, Viola thomasiana, Viscum album* |
|  | Sphagno-Piceetum | ***Picea abies****,* ***Vaccinium myrtillus****, Avenella flexuosa, Betula pubescens, Eriophorum vaginatum, Listera cordata, Lycopodium annotinum, Molinia caerulea, Pinus mugo, Sorbus aucuparia, Vaccinium uliginosum, Vaccinium vitis-idaea* |
|  | Erico-Pinion mugo/uncinatae | ***Erica carnea****,* ***Picea abies****,* ***Pinus mugo subsp. uncinata****,* ***Rhododendron hirsutum****, Arctostaphylos alpine, Calamagrostis varia, Carduus defloratus, Coronilla vaginalis, Crepis alpestris, Daphne striata, Gymnadenia odoratissima, Huperzia selago, Larix decidua, Leontodon incanus, Melampyrum sylvaticum, Pinus mugo, Polygala chamaebuxus, Pyrola media, Rhododendron ferrugineum, Rubus saxatilis, Sorbus chamaemespilus, Vaccinium gaultherioides, Vaccinium myrtillus, Vaccinium vitis-idaea* |
|  | Abieti-Piceion | ***Abies alba****,* ***Picea abies****,* ***Vaccinium myrtillus****, Adenostyles alliariae, Adenostyles glabra, Aposeris foetida, Asplenium viride, Athyrium filix-femina, Blechnum spicant, Calamagrostis varia, Circaea alpine, Dryopteris dilatata, Dryopteris expansa, Dryopteris filix-mas, Equisetum sylvaticum, Galium rotundifolium, Laburnum alpinum, Lamium galeobdolon, Lonicera alpigena, Lonicera nigra, Luzula sieberi, Lysimachia nemorum, Maianthemum bifolium, Melampyrum sylvaticum, Moneses uniflora, Oxalis acetosella, Petasites albus, Phyteuma spicatum, Prenanthes purpurea, Primula elatior, Pulmonaria mollis, Ranunculus serpens, Rhododendron ferrugineum, Saxifraga cuneifolia, Solidago virgaurea, Sorbus aucuparia, Valeriana tripteris, Veronica urticifolia* |
|  | Vaccinio-Piceion | ***Calamagrostis villosa****,* ***Picea abies****,* ***Vaccinium myrtillus****, Galium triflorum, Adenostyles alliariae, Athyrium distentifolium, Avenella flexuosa, Blechnum spicant, Cicerbita alpine, Corallorhiza trifida, Homogyne alpine, Larix decidua, Linnaea borealis, Listera cordata, Lonicera caerulea, Luzula luzulina, Luzula nivea, Luzula sieberi, Lycopodium annotinum, Melampyrum sylvaticum, Melica nutans, Oreopteris limbosperma, Orthilia secunda, Oxalis acetosella, Pyrola rotundifolia, Rhododendron ferrugineum, Saxifraga cuneifolia, Sorbus aucuparia, Vaccinium vitis-idaea, Veronica urticifolia* |
|  | Larici-Pinetum cembrae | ***Larix decidua****,* ***Rhododendron ferrugineum****,* ***Vaccinium gaultherioides****,* ***Vaccinium myrtillus****, Pinus cembra, Arctostaphylos uva-ursi, Avenella flexuosa, Calamagrostis villosa, Clematis alpine, Cotoneaster integerrimus, Homogyne alpine, Juniperus communis, Linnaea borealis, Lonicera caerulea, Luzula sieberi, Lycopodium annotinum, Melampyrum sylvaticum, Picea abies, Sorbus aucuparia, Vaccinium vitis-idaea* |
|  | Junipero-Laricetum/Mélézin | ***Larix decidua****,* ***Rhododendron ferrugineum****,* ***Vaccinium myrtillus****,* ***Vaccinium vitis-idaea,*** *Avenella flexuosa, Erica carnea, Festuca rubra, Homogyne alpine, Nardus stricta, Picea abies, Pulsatilla alpine****,*** *Rhododendron hirsutum* |
|  | Epilobion angustifolii | ***Epilobium angustifolium****, Digitalis purpurea, Senecio sylvaticus, Calamagrostis epigejos, Carex pilulifera, Galeopsis tetrahit, Gnaphalium sylvaticum, Holcus mollis, Lupinus polyphyllus, Mycelis muralis, Myosotis sylvatica, Rubus hirtus, Solidago virgaurea, Stellaria nemorum* |
| broadleaf | Alnion glutinosae | ***Alnus glutinosa****, Carex elongate, Dryopteris cristata, Galium elongatum, Hypericum androsaemum, Osmunda regalis, Ribes nigrum, Thelypteris palustris, Calamagrostis canescens, Carex acutiformis, Carex elata, Carex riparia, Dryopteris carthusiana, Equisetum fluviatile, Iris pseudacorus, Poa trivialis, Salix cinerea* |
|  | Salicion albae | ***Salix alba****, Salix fragilis, Salix viminalis, Humulus lupulus, Impatiens glandulifera, Leucojum aestivum, Myosoton aquaticum, Phalaris arundinacea, Phragmites australis, Populus alba, Populus nigra, Salix myrsinifolia, Salix purpurea, Salix triandra, Scutellaria hastifolia, Solanum dulcamara, Symphytum officinale, Urtica dioica, Viburnum opulus* |
|  | Alnion incanae | ***Alnus incana****,* ***Equisetum hyemale****,* ***Rubus caesius****, Aconitum variegatum, Aegopodium podagraria, Angelica sylvestris, Brachypodium sylvaticum, Calamagrostis epigejos, Calamagrostis varia, Caltha palustris, Carex acutiformis, Chrysosplenium alternifolium, Cornus sanguinea, Deschampsia cespitosa, Elymus caninus, Equisetum pretense, Equisetum sylvaticum, Euonymus europaeus, Festuca gigantean, Fraxinus excelsior, Humulus lupulus, Listera ovata, Lysimachia vulgaris, Malus sylvestris, Petasites albus, Petasites hybridus, Populus alba, Populus nigra, Prunus padus, Salix myrsinifolia, Salix pentandra, Salix purpurea, Solidago gigantean, Stachys sylvatica, Stellaria nemorum, Ulmus glabra, Urtica dioica, Viola biflora* |
|  | Luzulo-Fagenion | ***Fagus sylvatica****,* ***Luzula luzuloides****,* ***Luzula nivea****,* ***Luzula sylvatica****,* ***Melampyrum pratense****,* ***Quercus petraea****, Avenella flexuosa, Calamagrostis arundinacea, Calamintha grandiflora, Hieracium murorum, Ilex aquifolium, Monotropa hypophegea, Pinus sylvestris, Prenanthes purpurea, Pteridium aquilinum, Vaccinium myrtillus, Veronica officinalis* |
|  | Lunario-Acerion | ***Acer pseudoplatanus****,* ***Mercurialis perennis****, Aconitum variegatum, Campanula latifolia, Lunaria rediviva, Phyllitis scolopendrium, Polystichum setiferum, Acer platanoides, Aconitum vulparia, Actaea spicata, Anthriscus nitida, Arum maculatum, Aruncus dioicus, Cardamine pentaphyllos, Chrysosplenium alternifolium, Circaea x intermedia, Corydalis cava, Corydalis intermedia, Dryopteris filix-mas, Dryopteris remota, Fraxinus excelsior, Galium odoratum, Geranium robertianum, Impatiens noli-tangere, Lamium galeobdolon, Leucojum vernum, Paris quadrifolia, Polystichum aculeatum, Ranunculus lanuginosus, Ribes alpinum, Salvia glutinosa, Stellaria nemorum, Tilia platyphyllos, Ulmus glabra, Urtica dioica, Viola biflora* |
|  | Tilion platyphylli | ***Acer opalus****,* ***Corylus avellana****,* ***Mercurialis perennis****,* ***Tilia cordata****,* ***Tilia platyphyllos****, Euonymus latifolius, Staphylea pinnata, Acer platanoides, Acer pseudoplatanus, Asplenium trichomanes, Campanula rapunculoides, Clematis vitalba, Fraxinus excelsior, Galanthus nivalis, Geranium lucidum, Geranium robertianum, Gymnocarpium robertianum, Helleborus foetidus, Hepatica nobilis, Hippocrepis emerus, Ilex aquifolium, Poa nemoralis, Salvia glutinosa, Tamus communis, Ulmus glabra, Vincetoxicum hirundinaria, Viola mirabilis, Vitis sylvestris* |
|  | Carpinion | ***Anemone nemorosa****,* ***Carex montana, Carex pilosa, Carpinus betulus, Festuca heterophylla, Quercus petraea, Quercus robur,*** *Dactylis polygama, Isopyrum thalictroides, Stellaria holostea, Acer campestre, Acer opalus, Asarum europaeum, Brachypodium sylvaticum, Carex fritschii, Carex umbrosa, Corydalis solida, Cruciata glabra, Fraxinus excelsior, Galium aristatum, Galium laevigatum, Galium sylvaticum, Hedera helix, Knautia drymeia, Lathyrus linifolius, Lathyrus niger, Lonicera periclymenum, Luzula forsteri, Malus sylvestris, Melampyrum nemorosum, Melica uniflora, Mespilus germanica, Narcissus pseudonarcissus, Orobanche hederae, Poa nemoralis, Potentilla micrantha, Potentilla sterilis, Primula acaulis, Prunus avium, Pulmonaria montana, Pulmonaria officinalis, Pyrus pyraster, Ranunculus auricomus, Rosa arvensis, Scilla bifolia, Sorbus torminalis, Tilia cordata, Vinca minor, Viola alba, Viola riviniana* |
|  | Quercion pubescenti-petraeae | ***Acer opalus****,* ***Carex montana****,* ***Cornus mas****,* ***Hippocrepis emerus****,* ***Quercus petraea****,* ***Quercus pubescens****, Asplenium onopteris, Buglossoides purpurocaerulea, Lonicera etrusca, Mercurialis ovata, Viola suavis, Arabis turrita, Asplenium adiantum-nigrum, Brachypodium pinnatum, Buxus sempervirens, Campanula persicifolia, Carex halleriana, Carex humilis, Daphne laureola, Fraxinus excelsior, Helleborus foetidus, Hieracium glaucinum, Juniperus communis, Lathyrus niger, Limodorum abortivum, Melittis melissophyllum, Orchis mascula, Potentilla micrantha, Primula veris, Prunus mahaleb, Rhamnus alpine, Rhamnus cathartica, Ruscus aculeatus, Sesleria caerulea, Sorbus aria, Sorbus domestica, Sorbus torminalis, Tanacetum corymbosum, Teucrium chamaedrys, Viola alba* |
|  | Orno-Ostryon | ***Cornus mas****,* ***Fraxinus ornus****,* ***Hippocrepis emerus****,* ***Ostrya carpinifolia****,* ***Quercus pubescens****,* ***Teucrium chamaedrys****, Celtis australis, Cnidium silaifolium, Lathyrus venetus, Asparagus tenuifolius, Carex humilis, Cyclamen purpurascens, Daphne laureola, Helleborus niger, Hieracium glaucinum, Juniperus communis, Laburnum anagyroides, Lamium galeobdolon, Paeonia officinalis, Quercus cerris, Ruscus aculeatus, Sorbus aria, Tanacetum corymbosum* |
|  | Quercion robori-petraeae | ***Festuca heterophylla****,* ***Luzula nivea****,* ***Molinia arundinacea****,* ***Pteridium aquilinum****,* ***Quercus petraea****, Anthoxanthum odoratum, Avenella flexuosa, Betula pendula, Calamagrostis arundinacea, Calluna vulgaris, Carex montana, Dryopteris carthusiana, Euphrasia cisalpine, Hieracium glaucinum, Hieracium lachenalii, Hieracium laevigatum, Hieracium sabaudum, Hieracium umbellatum, Holcus mollis, Hypericum pulchrum, Lathyrus linifolius, Lonicera periclymenum, Luzula forsteri, Luzula luzuloides, Luzula pilosa, Melampyrum pretense, Phyteuma betonicifolium, Prunus serotine, Quercus robur, Sorbus torminalis, Teucrium scorodonia, Vaccinium myrtillus, Veronica officinalis Viola riviniana* |
|  | Castanea sativa forest | ***Castanea sativa****,* ***Festuca heterophylla****,* ***Luzula nivea****,* ***Molinia arundinacea****,* ***Pteridium aquilinum****,* ***Quercus petraea****, Anemone nemorosa, Anthoxanthum odoratum, Avenella flexuosa, Betula pendula, Calamagrostis arundinacea, Calluna vulgaris, Carex montana, Hieracium lachenalii, Hieracium laevigatum, Hieracium sabaudum, Hieracium umbellatum, Holcus mollis, Hypericum pulchrum, Lathyrus linifolius, Luzula forsteri, Luzula luzuloides, Luzula pilosa, Melampyrum pretense, Orchis mascula, Phyteuma betonicifolium, Quercus robur, Sorbus torminalis, Teucrium scorodonia, Vaccinium myrtillus, Veronica officinalis* |
|  | Betulion pubescentis | ***Betula pendula****,* ***Betula pubescens****,* ***Molinia caerulea****,* ***Vaccinium myrtillus****, Anthoxanthum odoratum, Avenella flexuosa, Calluna vulgaris, Dryopteris carthusiana, Frangula alnus, Lycopodium annotinum, Pinus mugo, Pinus sylvestris, Salix aurita, Salix cinerea, Vaccinium uliginosum* |
|  | Fraxinion | ***Fraxinus excelsior****,* ***Quercus robur****, Carex brizoides, Carex pendula, Carex remota, Carex strigose, Equisetum telmateia, Malaxis monophyllos, Matteuccia struthiopteris, Prunus padus, Ribes rubrum, Ulmus laevis, Adoxa moschatellina, Aesculus hippocastanum, Alnus glutinosa, Alnus incana, Anemone nemorosa, Anemone ranunculoides, Arum maculatum, Carex acutiformis, Chrysosplenium alternifolium, Circaea lutetiana, Clematis vitalba, Equisetum hyemale, Equisetum sylvaticum, Festuca gigantea, Gagea lutea, Juglans regia, Lamium galeobdolon, Lathraea squamaria, Leucojum vernum, Listera ovata, Lysimachia nemorum, Lysimachia nummularia, Platanus orientalis, Poa remota, Populus alba, Primula elatior, Ranunculus ficaria, Rumex sanguineus, Scilla bifolia, Scrophularia nodosa, Stachys sylvatica, Stellaria nemorum, Ulmus minor, Veronica hederifolia, Veronica montana, Viburnum opulus* |
|  | Galio-Fagenion | ***Allium ursinum****,* ***Anemone nemorosa****,* ***Arum maculatum****,* ***Circaea lutetiana****,* ***Fagus sylvatica****,* ***Galium odoratum****, Cardamine bulbifera, Carex digitate, Carex sylvatica, Carpinus betulus, Dryopteris filix-mas, Epilobium montanum, Euphorbia dulcis, Fraxinus excelsior, Hedera helix, Lamium galeobdolon, Lathyrus vernus, Lonicera xylosteum, Luzula pilosa, Mercurialis perennis, Paris quadrifolia, Polygonatum multiflorum, Prenanthes purpurea, Primula acaulis, Pulmonaria obscura, Pulmonaria officinalis, Quercus petraea, Quercus robur, Sanicula europaea, Viola reichenbachiana* |
|  | Atropion | ***Fragaria vesca****,* ***Galeopsis tetrahit****,* ***Rubus idaeus****, Arctium nemorosum, Bromus ramosus, Geranium bohemicum, Hypericum hirsutum, Stachys alpine, Atropa bella-donna, Calamagrostis epigejos, Carex divulsa, Carex guestphalica, Carex spicata, Digitalis lutea, Eupatorium cannabinum, Mycelis muralis, Sambucus ebulus, Senecio ovatus, Verbascum nigrum, Verbascum thapsus* |
| mixed | Cephalanthero-Fagenion | ***Acer opalus****,* ***Carex alba,*** ***Carex flacca****,* ***Carex montana****,* ***Fagus sylvatica****,* ***Quercus petraea****,* ***Sesleria caerulea****,* ***Taxus baccata****, Cephalanthera damasonium, Cephalanthera longifolia, Cephalanthera rubra, Acer campestre, Aster bellidiastrum, Brachypodium pinnatum, Calamagrostis varia, Campanula persicifolia, Convallaria majalis, Cyclamen purpurascens, Daphne mezereum, Doronicum pardalianches, Epipactis helleborine, Epipactis microphylla, Helleborus viridis, Hepatica nobilis, Hippocrepis emerus, Ligustrum vulgare, Melittis melissophyllum, Mercurialis perennis, Neottia nidus-avis, Pinus sylvestris, Rhamnus alpine, Rubus saxatilis, Sorbus aria, Viburnum lantana, Vincetoxicum hirundinaria* |
|  | Dicrano-Pinion | ***Calluna vulgaris****,* ***Pinus sylvestris****,* ***Vaccinium myrtillus,*** *Chimaphila umbellate, Diphasiastrum complanatum, Avenella flexuosa, Betula pendula, Calamagrostis villosa, Carex ericetorum, Frangula alnus, Juniperus communis, Melampyrum pretense, Orthilia secunda, Picea abies, Pteridium aquilinum, Quercus petraea, Quercus robur, Rumex acetosella, Vaccinium vitis-idaea* |
|  | Ledo-Pinion/Piceo-Vaccinienion uliginosi | ***Pinus mugo subsp. uncinata****,* ***Vaccinium myrtillus****,* ***Vaccinium uliginosum****, Andromeda polifolia, Betula nana, Betula pubescens, Calluna vulgaris, Carex nigra, Carex rostrate, Eriophorum vaginatum, Lycopodium annotinum, Molinia caerulea, Picea abies, Sorbus aucuparia, Trichophorum cespitosum, Vaccinium oxycoccos, Vaccinium vitis-idaea* |
|  | Lonicero-Fagenion | ***Fagus sylvatica****,* ***Galium odoratum****,* ***Lamium galeobdolon subsp. Montanum****,* ***Mercurialis perennis****,* *Cardamine heptaphylla*, *Abies alba*, *Acer pseudoplatanus*, *Adenostyles glabra*, *Cardamine kitaibelii*, *Cardamine pentaphyllos*, *Cardamine trifolia*, *Carex sylvatica*, *Daphne mezereum*, *Dryopteris affinis*, *Euphorbia amygdaloides*, *Gymnocarpium dryopteris*, *Heracleum sphondylium*, *Hordelymus europaeus*, *Laburnum alpinum*, *Lathyrus vernus*, *Lilium martagon*, *Lonicera alpigena*, *Lonicera nigra*, *Melica uniflora*, *Milium effusum*, *Oxalis acetosella*, *Phyteuma spicatum*, *Picea abies*, *Polygonatum verticillatum*, *Prenanthes purpurea*, *Tilia platyphyllos*, *Veronica urticifolia*, *Viola reichenbachiana* |
|  | Abieti-Fagenion | ***Abies alba****,* ***Adenostyles alliariae****,* ***Athyrium filix-femina****,* ***Fagus sylvatica****,* ***Hordelymus europaeus****,* ***Picea abies****, Acer pseudoplatanus, Aconitum vulparia, Adenostyles glabra, Cardamine pentaphyllos, Cicerbita alpine, Dryopteris filix-mas, Festuca altissima, Galium rotundifolium, Geranium sylvaticum, Gymnocarpium dryopteris, Hieracium murorum, Laburnum alpinum, Lamium galeobdolon, Lonicera nigra, Luzula sylvatica, Milium effusum, Oxalis acetosella, Paris quadrifolia, Petasites albus, Phegopteris connectilis, Phyteuma spicatum, Polygonatum verticillatum, Polystichum aculeatum, Prenanthes purpurea, Primula elatior, Ranunculus aconitifolius, Ranunculus lanuginosus, Rumex alpestris, Saxifraga rotundifolia, Streptopus amplexifolius, Thalictrum aquilegiifolium, Vaccinium myrtillus, Viola reichenbachiana, Viscum album* |
|  |  |  |
