# Supplementary material for: Local and landscape-level diversity effects on forest functioning
Source: PLoS One. 2020 May 14;15(5):e0233104. doi: 10.1371/journal.pone.0233104 (PMC7224498; doi:10.1371/journal.pone.0233104)
Supplement: S2 Table — List of all the tree species we found in the tree inventory, the number of individuals across all study sites and number of study sites with a respective species present. (DOCX) [file pone.0233104.s004.docx]

|  |  |  |
| --- | --- | --- |
| **Species** | **Number of individuals** | **Number of study sites** |
|  |  |  |
| *Abies alba* | 99 | 13 |
| *Acer campestre* | 13 | 3 |
| *Acer platanoides* | 2 | 1 |
| *Acer pseudoplatanus* | 188 | 9 |
| *Alnus incana* | 81 | 1 |
| *Alnus viridis* | 8 | 2 |
| *Betula pendula* | 82 | 8 |
| *Carpinus betulus* | 1 | 1 |
| *Castanea sativa* | 126 | 3 |
| *Cornus mas* | 1 | 1 |
| *Corylus avellana* | 99 | 5 |
| *Fagus sylvatica* | 346 | 19 |
| *Fraxinus excelsior* | 89 | 14 |
| *Juglans regia* | 16 | 1 |
| *Juniperus communis* | 1 | 1 |
| *Larix decidua* | 38 | 10 |
| *Picea abies* | 664 | 26 |
| *Pinus cembra* | 35 | 2 |
| *Pinus sylvestris* | 56 | 2 |
| *Populus tremula* | 23 | 3 |
| *Prunus avium* | 68 | 8 |
| *Pyrus pyraster* | 1 | 1 |
| *Quercus petrea* | 25 | 5 |
| *Quercus pubescens* | 29 | 1 |
| *Quercus robur* | 5 | 2 |
| *Salix caprea* | 15 | 6 |
| *Sorbus aria* | 22 | 3 |
| *Sorbus aucuparia* | 144 | 5 |
| *Taxus baccata* | 2 | 2 |
| *Tilia cordata* | 23 | 1 |
| *Tilia platyphyllos* | 17 | 2 |
| *Ulmus glabra* | 15 | 3 |
| *Ulmus minor* | 7 | 1 |
|  |  |  |
| Total number of trees in inventory | 2341 |  |
