# Supplementary material for: Local and landscape-level diversity effects on forest functioning
Source: PLoS One. 2020 May 14;15(5):e0233104. doi: 10.1371/journal.pone.0233104 (PMC7224498; doi:10.1371/journal.pone.0233104)
Supplement: Attachment — Submitted filename: Response_to_Reviewers_PLOS_Oehri.docx [file pone.0233104.s006.docx]

April 18, 2020

Response to Reviewers

We thank both reviewers for their constructive critique that enabled us to improve our manuscript. We address the raised issues in a point-by-point response to the reviewers’ comments below. For better readability, we write using blue font. Line numbers refer to the unmarked version of the revised manuscript.

Yours sincerely,

Jacqueline Oehri (on behalf of all authors)

**Reviewer #1:** This is a well-conceived study; I like the idea of testing ideas about richness effects on ecosystem functioning in real world landscapes. In general, the study was designed well, but I am concerned that some of the models may be overfit, given the small sample size and number of predictor variables.

The manuscript was very well written and easy to understand in most places. The English expression is good but could be improved in a few spots (I haven’t made any comments about this).

Below are some specific comments

Thank you. We agree and address your concerns as described below. We also improved the English expressions in a few spots including the ones recommended by reviewer #2.

Line 104. I don’t understand the reference to Jaccard similarities – what do they have to do with the vegetation communities outlined in Table S1?

Thank you for pointing this out. This was indeed not well explained. We now add a more detailed explanation on lines 117 ff. For more clarity, we also replaced the “dominant species” lists with the full, “characteristic species” list that we used to calculate Jaccard indices for each of the 30 forest types in S1 Table.

Specifically, we explain that for all 1416 sites monitored by the Swiss biodiversity monitoring program (BDM), we derived their respective similarity to the 30 typical forest communities in Switzerland (S1 Table) by using Jaccard’s similarity index (Jaccard 1912): J=$\frac{|A \cap B|}{|A \cup B|}$, where A and B are two sample sets and 0 $\leq$ J(A,B) $\leq$ 1. In our case, A referred to the lists of species names monitored for each of the 1416 BDM sites, and B referred to the lists of characteristic species names for the 30 typical forest communities in Switzerland (S1 Table). This procedure enabled us to estimate the likelihood of each of the 1416 BDM-sites to be a coniferous, mixed or broadleaf forest (or to be a forest at all).

Jaccard, P. The distribution of the flora in the alpine zone. *New Phytologist*; 1912, 11, 37–50.

Line 140. If you’ve used average altitude doesn’t the variable reflect average altitude rather than topography (ie, I’m suggesting perhaps renaming it mean altitude)

Indeed, this was not described with enough clarity. The actual lines you refer to (now lines 157 ff.) say “We characterized landscape-level topography by mean altitude (...), slope (…) and northerly aspect of the slope (…).”, therefore we leave the expression of topography there.

However, it is true that in the main part of our analysis, we focused on mean altitude as a predictor and therefore, the notion of topography might be misleading. What we did in our study was first assessing several candidate variables for each environmental category (stand structure, landscape structure, climate and topography) and then selecting one representative variable for each category. We did this in order to reduce multicollinearity among explanatory variables.

We now state our procedures more clearly in the Introduction (lines 96 ff.) & Materials and methods (lines 206 ff.). Also, to further improve the clarity we supplemented the notion of “topography” with “mean altitude” in the Abstract and Discussion sections.

Line 180-181. Is the PCA necessary? Does it add anything?

Thank you for pointing this out. It is true that we used the PCA mainly as an additional tool to confirm our predictor selection. Specifically, we mainly used it to highlight and substantiate the “clustering” i.e. positive/negative covariation among predictors (shown by the angle among predictor’s arrows in S2 Fig [angles around +/-90° indicate weak covariation]).

However, we also used it to 2) reveal the important environmental gradients in our dataset (shown by the “loadings” of the predictors on the first two principal component axes PC1 and PC2 in S2 Fig [i.e. the projection of the predictor’s arrows onto PC1 or PC2]). Therefore, the PCA was nevertheless a valuable tool to select a set of explanatory variables that represented important environmental gradients and that had reduced multicollinearity.

Because the PCA was an integral part of our study procedure, we think it is more transparent to describe it in the manuscript. We clarified the description on lines 206 ff.

Line 183-185. I’d suggest using a model with poisson errors, as species richness is a count. Then your inferences are about species richness rather than log(SR). If you use a poisson model check for overdispersion.

Thank you for pointing this out. This was a misleading description on our part, which understandably raises the issue you mention.

Indeed, it would be more appropriate to use a model with poisson errors if we would model species richness as a dependent variable. However, we use species richness as an independent variable to predict the continuous dependent variables LAI and GSL. Therefore, a model with poisson errors would not be suited for our analysis.

We now corrected our previous misleading statement: because BEF research has found that ecosystem functions typically increase linearly with the logarithm of species richness, we investigated models with untransformed and log-transformed species richness. Model fits and species richness effects were similar but there was some indication that the log-transformation did linearize relationships, as found in the cited previous papers, coming mainly from experiments. We therefore decided to show results for log-transformed species richness for better comparability with BEF studies.

We changed the description in the Materials and methods section accordingly (lines 216 ff.).

Lines 195-210. It seems that at least some of your models contain all eight predictor variables. With a sample size of 36 (and I think 22 for GSL – line 233 – although I missed this in the methods) your models will be overfitted. A common rule of thumb is one estimated parameter for each 10 data points. While I think we can push this a bit, with 36 data points you could perhaps build a model with 4 or 5 continuous predictors. And, because BGR and FT are categorical, they make this problem worse. For example, BGR has 6 categories, so 5 parameters are estimated by the regression model. Ideally we’d like 40-50 samples to build a model containing only BGR. Unless I’ve misunderstood how you’ve analysed your data I fear your sample size is inadequate for the complexity of your models. Perhaps you could achieve your objectives using simpler models.

This is indeed a very good and important point.

1) The previous results from the variance partitioning analysis (previously reported in Fig 3) were indeed based on models with all 8 predictors. We have now changed this analysis and show results of models with all possible combinations of either 1, 2 or 3 of the selected 8 predictors (Fig 3).

The findings of the new variance partitioning analysis remain relatively similar as reported in the previous version of the manuscript. We changed the manuscript accordingly in the Materials and methods (lines 226 ff.) and Results (lines 286 ff.) section.

2) In the general linear models summarized by ANOVA (see Table 1 for an overview), we actually did not use all 8 predictors simultaneously. As indicated in the “model terms” column in Table 1 the models either used one, two or three out of the 8 predictors.

We now explain this more clearly in the Materials and methods sections (lines 233 ff.) & Result sections (lines 328 ff.) and in the legend of Table 1.

3) We acknowledge that the models with biogeographic region (BGR) and forest type (FT) actually fit more than 3 contrasts. However, BGR and FT both represent study design variables with systematic effects on our dependent variables LAI and GSL. Therefore, results from models that account for these design variables before testing effects of other predictors are a valid and important part of the evidence. We tackle the trade-off between model over- and under-fitting by reporting on an array of models with different predictor combinations and model-complexities. Our main finding from our analysis is based on the entirety of these models taken together; and is that species richness effects on LAI and GSL are relatively significant and similar in size across all models.

Line 212-221. If this section is about correlations among predictors (as the sub-heading suggests, remove LAI and GSL, as these are your response variables). As a general comment, although it’s interesting to know which of your original suite of predictors correlated with each other, I think it is your least important result, and I suggest putting it last in the results section. Lead with your most interesting result. If you proceed with reordering your sections also do so in the methods and discussion.

Thank you for this comment. We agree that this is the least interesting result. Nevertheless, we found that because this analysis represents the basis of all further analyses in the manuscript, a relocation towards the end of Methods/Results/Discussion sections impairs the clarity of our manuscript. Therefore, we think it is more appropriate to leave the topic at the current position in the text. Also, in our opinion, it serves a better understanding of our results to leave in the ecosystem functioning variables in the Figure (Fig 2).

We therefore added “ecosystem functions” in the heading of this section (line 243).

Line 258-259. I’m not sure what you mean by ‘ranged among the most important predictors’.
Thank you for your comment; this was indeed an unclear statement. We meant that species richness *was* among the most important predictors; because it explained the second highest amount of variance in leaf area index (only biogeographic region explained more variance).

We now changed the wording in the manuscript where appropriate (i.e. in the Abstract, the Results and Discussion; lines 41, 291 and 370, respectively).

Line 335-338. You could answer this question using some kind of network analysis (eg a structured equation model or a Bayesian network).

Thank you for this nice suggestion. We indeed explored the possibility of applying such network analyses (see Fig R1 below).

However, unfortunately, because of the limited amount of study units, the amount of paths that can be modelled with these analyses is limited, too. In our case, appropriate models would be restricted to three paths among the three variables land-cover richness, species richness and ecosystem functioning.

Applying a relatively simple, three-path structural equation model, for example, is mathematically not so different from our general linear model analyses summarized in Table 1; where we assessed land-cover richness effects when fitted before and after species richness. Additionally, correcting for study design variables such as biogeographic region is not trivial in such network analyses, and in our case not applicable because of the small number of study units.

Hence, we could not apply a network analysis with appropriate complexity to adequately assess the indirect effects of land-cover richness on ecosystem functions. Therefore, we stay with the previous statements. However, we expand our explanation of potential mechanisms in the Discussion on lines 434 ff.

**Fig R1. Indirect effects of landscape richness via species richness**. Using structural equation modeling (SEM; Grace 2006) and the lavaan R-package (Rosseel 2012) we assessed the indirect landscape richness (LR) effects on forest functioning mediated by tree species richness (log(SR)). Solid lines represent positive effects; dashed lines represent negative effects; line width increases with effect size. Standardized path coefficient estimates and their significances (‘**’:P<0.01; ‘*’:P<0.05; ‘.’: P<0.1; ‘n.s.’: not significant) are shown next to the corresponding paths. The letter zeta refers to the residual error, i.e. variance in forest functions not explained by the SEM. The goodness of fit is shown by the chi-square test.; comparative fit index CFI and standardized root mean square residual SRMR. Number of study units n= 36 for LAI and n=22 for GSL. **A:** SEM with direct and indirect effects of LR on LAI, standardized indirect effect via log(SR) [mean±s.e.] β=0.17 ± 0.09, P=0.07. **B:** SEM with an indirect LR effect only, standardized indirect effect [mean±s.e.] β=0.14 ± 0.08, P=0.08, **C:** SEM with direct and indirect effects of LR on GSL, standardized indirect effect via log(SR) [mean±s.e.] β=0.07 ± 0.09, P=0.4. **D:** SEM with an indirect LR effect only, standardized indirect effect [mean±s.e.] β=0.07 ± 0.09, P=0.4.

Grace JB. Structural equation modeling and natural systems: Cambridge University Press; 2006.

Rosseel Y. lavaan: An R Package for Structural Equation Modeling. Journal of Statistical Software. 2012;48(2):1-36.

Line 380-389. I don’t understand your point about a scale mismatch. Isn’t the idea behind this kind of analysis that landscape patterns influence processes occurring at a point? Perhaps I’m misunderstanding your meaning.

Thank you for pointing this out. We agree and find that this paragraph might be more confusing than contributing to a better understanding. Therefore, we deleted this paragraph.

**Reviewer #2:** The manuscript "Local and landscape-level diversity effects on forest functioning" by Oehri et al. presents an assessment of the relationship between tree species richness and landcover-type richness on two measures of forest ecosystem function, leaf area index and growing season length, within 36 forest stands in Switzerland. The design of the study and the analyses of the data appear to be appropriate. The manuscript is well-written and describes results that provide further evidence of the importance of biodiversity to the functioning of forested ecosystems.

The manuscript has a few relatively minor shortcomings that should be addressed by the authors.

Thank you very much, please find our comments below.

First, the Introduction could be improved by including a more specific statement of the hypotheses and/or objectives of the study. As written, the manuscript comes across as being somewhat descriptive, but I don't think that was the intent of the authors. Listing specific hypotheses in the Introduction and then addressing in the Discussion whether the results support these hypotheses would make for a stronger manuscript.

Thank you for this comment. We agree that adding our hypotheses would increase the clarity of the manuscript and therefore added specific hypothesis statements at the end of the Introduction section on lines 103 ff. We discuss if hypotheses are supported at the start of the Discussion section on lines 374 ff.

Second, I could not find an explanation for why the authors choose these particular ecosystem functions and indicators for this study. Why are these functions important, and why use these specific indicators to assess these functions?
Thank you for pointing this out. We now added an explanation of the relevance of these particular ecosystem functions as well as the respective ways to approximate them in the Introduction on lines 85 ff. and in the Materials and methods section on lines 167 ff. and 178 ff.

Third, some important details are missing that would help readers to better understand the methods.

For example, how were the locations of the sites determined? The caption for figure 1 (lines 109-110) says they were "regularly spread" across the country, but how?

We agree that our explanations were not clear enough and therefore added clarifications in the caption of Fig 1 (lines 126 ff.) and in the Materials and methods section (lines 113 ff.).

Specifically, the locations of the 1416 biodiversity monitoring (BDM) sites are pre-determined by the BDM and correspond to the small black dots shown in Fig 1. The BDM deliberately selected these sites based on site-coordinates so that they are equally spaced within the Swiss CH1903 coordinate system. More details on how the BDM selected sites are available in (BDM 2011).

We selected the subset of 36 forest sites out of the total 1416 BDM sites as follows: First, we quantified the similarity of the species lists available for all 1416 BDM sites and the species lists of the 30 typical forest communities in Switzerland (S1 Table) using Jaccard’s similarity index ((Jaccard 1912); see reviewer #1’s comment “Line 104” above). This procedure allowed us to classify each BDM site as “coniferous”, “mixed” or “broadleaf” (or “no”) forest. In each of the six biogeographic regions (BGR) of Switzerland, we then randomly selected two BDM sites classified as “coniferous forest”, two BDM sites classified as “mixed forest” and two BDM sites classified as “broadleaf forest” for further investigation.

BDM Coordination Office. Sample Design of Biodiversity Monitoring Switzerland (BDM). Federal Office for the Environment Bern. http://www.biodiversitymonitoring.ch/fileadmin/user_upload/documents/daten/anleitungen/950_Stichprobendesign_v4_En.pdf; 2011 pp. 1-20.

Jaccard, P. The Distribution of the flora in the alpine zone. *New Phytologist*; 1912, 11, 37–50.

How was peak-season LAI determined (line 150)? The total peak over the two years? Or the mean of the two years? Or something else? I get the sense that LAI measurements were taken once a year at each plot. How did the authors determine when they would collect these measurements?

Thank you for this comment. The expression of “peak-season” was misleading. We measured LAI once per summer in the time period when the forest canopy was fully developed, i.e. at a time well after leaf-out but also before leaf-fall, which is estimated to be between the end of April and the end of October, respectively, in the northern alpine foreland (Chmielewski and Rötzer 2001)). Specifically, the average time of LAI measurements was the end of August. We took LAI measurements at three random locations within the study plots in every year and then averaged these values first for each plot in each year. Finally, we averaged these values for each study plot across the two years.

We clarified the description of our method on lines 169 ff.

Chmielewski FM, Rötzer T. Response of tree phenology to climate change across Europe. Agricultural and Forest Meteorology. 2001;108(2):101-12.

How was principal component analysis (lines 180-181) used to verify the strengths of the correlations among the variables?

We used the PCA to confirm the relationships among predictors that we already identified using the overall correlation analysis (Fig 2). Specifically, we confirmed that positive and negative correlations among predictors from the correlation analysis were in accordance with clusters of predictors in the PCA, where positive & negative correlations are shown by the angle among predictor’s arrows (angles of around +/-90° indicate weak correlation; S2 Fig). Additionally, we used the PCA to see if selected predictors represented important environmental gradients in our dataset. The importance of predictors is shown by their “loadings” on the first two principal component axes PC1 and PC2, i.e. the projection of a predictor’s arrow onto PC1 or PC2.

Hence, the PCA was an integral part of our study procedure, and we clarified the description on lines 206 ff.

How were the GLMs conducted to account for the effect of biogeographic region (lines 205-207)?

We accounted for effects of biogeographic region (BGR) simply by fitting the term “BGR” before fitting the term “log(SR)” or “LR” in the models (i.e. the models were specified as “GSL ~ β_0_ + β_1_*BGR + β_2_*precip + β_3_*log(SR) + ε”, for example).

We made our methods clearer by adapting lines 234 ff.

Fourth, some elaboration in the Discussion would be helpful about the relationships between tree species richness, growing season length and biogeographic regions (lines 367-372). How was biogeographic region controlled for? Are biogeographic region and growing season length related in some way?

Thank you for this important question. As we describe above, we controlled (or accounted) for effects of biogeographic region by fitting biogeographic region as an explanatory variable before fitting the species richness term in our general linear models. Furthermore, we added now a more detailed description on how biogeographic region, growing season, altitude and species richness are related on lines 407 ff.

Interestingly, growing season strongly decreases with altitude, and tends to be lower in the Eastern and Western Central Alps compared to the rest of the biogeographic regions. Species richness also decreased with altitude but did not depend on biogeographic region. See Fig R2 for an overview:

**Fig R2. Relationships of growing season length (GSL), tree species richness (logSR) and mean altitude (alt). A**: Growing season length tended to be lower in the Eastern and Western Central Alps compared to the other biogeographic regions (F_5,16_=2.5, P=0.078). **B**: Tree species richness (log-transformed) was not dependent on biogeographic region (F_5,30_=0.5, P=0.8). **C**: Growing season length decreased with mean altitude (F_1,20_=45.0, P<0.001). **D**: Tree species richness (log-transformed) but correlated negatively with altitude (F_1,34_=11.1, P=0.002). Solid and dashed lines represent model-predicted mean ± s.e.m..

Additional comments

Abstract:

-- The abstract makes no mention about where this study was conducted. This should be mentioned (possibly near lines 33-35).

Yes, we agree, and added central Europe (Switzerland) as location.

-- Line 40: I don't think the word "ranges" works here. The word "is" works better.

Yes, we agree, and changed the sentence accordingly.

-- Lines 39-41: Is it worth mentioning that growing season length and and tree species richness may be confounded with altitude?

Following a suggestion of reviewer #1 (see above) we supplemented the general notion of “topography” with the more specific “mean altitude” on line 41. Because the sentence starts with “We then examined the correlation and the confounding of these (…)”, we think that it is now clear that there could be confounding between species richness and altitude (and also between all other mentioned variables).

Introduction:

-- Lines 52-54: This sentence is missing something.

We changed the wording to clarify the message of the sentence.

-- Line 53: What is a "positive decelerating response"? Is there a clearer way to state this?
We changed the wording to clarify the message of the sentence.

-- Lines 56-58: Note there have been a number of BEF studies in the United States and Canada using many thousands of plots in natural forest settings. See, for example:

Thank you for pointing these out. Indeed, they are important to include in our manuscript. We added Potter et al. 2014 and Iannone et al. 2016 at appropriate locations (see lines 402 and 456). (Paquette & Messier were already in there at line 402).

Paquette, A. and Messier, C. 2011. The effect of biodiversity on tree productivity: from temperate to boreal forests. Global Ecology and Biogeography, 20: 170-180. doi:10.1111/j.1466-8238.2010.00592.x

Potter, K.M., and C.W. Woodall. 2014. Does biodiversity make a difference? Relationships between species richness, evolutionary diversity, and aboveground live tree biomass across U.S. forests. Forest Ecology and Management. 321:117-129. <https://doi.org/10.1016/j.foreco.2013.06.026>.

Iannone, B.V., K.M. Potter, K-A. Dixon Hamil, W. Huang, H. Zhang, Q. Guo, C.M. Oswalt, C.W. Woodall, and S. Fei. 2016. Evidence of biotic resistance to invasions in forests of the Eastern USA. Landscape Ecology. 31:85-99. <https://doi.org/10.1007/s10980-015-0280-7>.

-- Lines 80ff: Articles are needed in several cases in this paragraph, such as "an indicator", "a measure", "the primary indicator", etc.

Thank you for pointing these out. We added these articles throughout the manuscript where appropriate.

Methods
-- Line 123: It's is probably worth noting here that the rarefied tree species richness was scaled to the plot with the smallest number of trees.

We changed the statement as suggested.

-- Line 163: "to only record" would be easier to read as "to record only"

We changed the statement as suggested.

-- Line 173: Should "about the threshold" be "above the threshold"?

Yes. Thank you, we changed the statement as suggested.

-- Line 179: I recommend adding "categorized as" before "small, medium and large"

Yes, we agree and changed the statement as suggested.

-- Line 184ff: "an" should be added before "indicator"

Yes, we added the article where appropriate.

-- Line 198: Would it be possible to add a citation for the variance partitioning method?
Yes, we have indicated two citations:

“(…) variance partitioning (method by [44] as implemented in the lmg function of the R library relaimpo [45])”

44.	Lindeman RH, Merenda PF, Gold RZ. Introduction to Bivariate and Multivariate Analysis. Glenview, IL: Scott Foresman; 1980.

45.	Grömping U. Relative Importance for Linear Regression in R: The Package relaimpo. Journal of Statistical Software. 2006;17(1):1-27.

Results

-- Line 215: I don't think the word "marginally" works here, because the correlation is actually fairly large while the level of significance is not. Something like "at a lower significance" or "at a higher P value"

Yes, this is a good point; we added the “at a lower significance” statement on line 246.

Discussion

-- Line 349: I would change "was" to "may have been"

Yes, we agree and changed description to “tended to being (…)” on line 392.

-- Line 362: "was" needed after "relationship"

Yes, we changed the sentence accordingly.

-- Lines 392-395: This is an interesting idea. What would the mechanism be?

Yes, indeed. We added a paragraph with potential mechanisms in the Discussion (lines 434 ff.).

-- Lines 404-406: Should this issue with coniferous forest plots be addressed in the methods or results?

Thank you for pointing at this. We think that this issue is given the proper context by being described in the Discussion section. Since our number of study units is fairly small and our variables are not fully orthogonal, results from testing interaction terms should not be given too much weight in our manuscript. However, we still wanted to report on the fact that we did not find any indication of significant interactions terms except in the cases where growing season length was ill-defined. Hence, the interaction-term analysis is a tentative, additional support for one of our main findings, namely that tree species diversity effects are rather similar across a range of environmental conditions.

-- Lines 413-415: I think this idea could be elaborated on further, either here or elsewhere in the Discussion.

Yes, we agree and added a paragraph elaborating on possible mechanisms on lines 434 ff. in the Discussion.

-- Line 418: "the" is needed before "effects"

Yes, we added this article accordingly.
